# Vaccine Hesitancy and Associated Factors Among Caregivers of Children With Special Health Care Needs in the COVID-19 Era in China: Cross-Sectional Study

**DOI:** 10.2196/67487

**Published:** 2025-03-26

**Authors:** Mingyan Li, Changxuan Sun, Chai Ji, Meiying Gao, Xia Wang, Dan Yao, Junxia Guo, Lidan Sun, Abdul Rafay, Antonita Shereen George, Sanduni Hasara Samararathna Samararathna Muhandiramge, Guannan Bai

**Affiliations:** 1 Department of Child Health Care, Children’s Hospital, Zhejiang University School of Medicine, National Clinical Research Center for Child Health, Hangzhou, China; 2 The Fourth Affiliated Hospital of Soochow University, Medical Center of Soochow University, Suzhou Dushu Lake Hospital, Suzhou, China; 3 School of Medicine, Imperial College London, London, United Kingdom; 4 First Affiliated Hospital of Zhejiang University, Zhejiang University School of Medicine, Hangzhou, China

**Keywords:** COVID-19, caregivers, children with special health care needs, vaccination hesitancy, decision-making

## Abstract

**Background:**

Immunization is a cost-effective way to prevent infectious diseases in children, but parental hesitancy leads to low vaccination rates, leaving children at risk. Caregivers of children with special health care needs are more hesitant about vaccines than those of healthy children.

**Objective:**

The aim of the study is to investigate the changes in caregivers’ vaccination hesitation of children with special health care needs before, during, and after the COVID-19 pandemic in China and to identify associated factors for caregivers’ attitudes toward National Immunization Program (NIP) and non-NIP vaccines.

**Methods:**

We included 7770 caregivers of children with special health care needs (median age 7.0, IQR 2.4-24.1 months) who visited the Vaccination Consultation Clinic at Children’s Hospital, Zhejiang University School of Medicine (Hangzhou, China) from May 2017 to May 2023. General and clinical information was extracted from the immunization evaluation system for children with special health care needs and medical records. We compared the differences in caregivers’ willingness and hesitation for vaccinating their children across the 3 stages of the COVID-19 pandemic using chi-square tests. Multinomial logistic regression models were used to identify independent variables that were associated with caregivers’ willingness and hesitation toward NIP and non-NIP vaccines.

**Results:**

There is a statistically significant difference in caregivers’ vaccine hesitancy before, during, and after the COVID-19 pandemic (*P*<.05). During the COVID-19 pandemic, the percentages of choosing NIP, alternative non-NIP, and non-NIP vaccines are highest (n=1428, 26%, n=3148, 57.4%, and n=3442, 62.7%, respectively) than those at other 2 stages. In comparison, caregivers’ hesitation toward NIP and non-NIP vaccines is lowest (n=911, 16.6% and n=2045, 37.3%, respectively). Despite the stages of the COVID-19 pandemic, multiple factors, including children’s age and sex, parents’ educational level, comorbidities, and history of allergy, were significantly associated with caregivers’ attitude toward NIP and non-NIP vaccines (*P*<.05). The profiles of risk factors for hesitancy toward NIP and non-NIP vaccines are different, as indicated by the results from the logistic regression models.

**Conclusions:**

This study demonstrated that caregivers’ willingness to vaccinate their children with special health care needs with NIP and non-NIP vaccines was highest during the COVID-19 pandemic in China, and their hesitancy was lowest. Additionally, we have identified multiple factors associated with caregivers’ willingness and hesitancy to vaccinate their children. These findings provide evidence-based support for developing personalized health education strategies.

## Introduction

Immunization is widely recognized as an exceptionally cost-effective strategy for preventing and eliminating infectious diseases in children. In China, a dual-tier vaccination program is implemented. The government provides vaccines under the National Immunization Program (NIP) at no cost across the nation, whereas non-NIP vaccines incur some costs [1]. It is mandatory for all children to receive NIP vaccines according to governmental guidelines. The NIP vaccines in China, which include 14 vaccines targeting 15 diseases, have achieved a coverage rate exceeding 95% among children younger than 6 years of age at the national level [2,3]. In contrast, non-NIP vaccines, which complement the NIP vaccines and are crucial for comprehensive disease prevention and control, exhibit relatively low coverage rates. These rates vary significantly across different regions and socioeconomic groups primarily because non-NIP vaccines are voluntary, self-funded, and not required for school enrollment. Zhou et al [4] observed that the coverage of non-NIP vaccines was lowest among left-behind families and highest among local urban families. Generally, the coverage rates for most non-NIP vaccines are below 50%, with specific rates ranging from 1.8% for the third dose of the rotavirus vaccine to 67.1% for the first dose of the varicella vaccine [5].

Numerous factors can affect vaccine coverage rates. Studies demonstrate a direct correlation between caregivers’ attitudes toward vaccines and vaccination rates [6]. A significant number of caregivers express hesitancy regarding vaccinating their children often due to concerns about safety, perceived inconvenience, financial costs [7], and a lack of trust in scientific evidence [8]. This hesitancy results in insufficient vaccination coverage and leaves children vulnerable to vaccine-preventable diseases [9]. Notably, caregivers of children with special health care needs are more hesitant about vaccines than those of healthy children [10]. Children with special health care needs refers to a population of children who either have or are at an elevated risk for chronic physical, developmental, behavioral, or emotional conditions. In China, about 2.12 million newborns face birth defects or immune issues each year [11]. Recently, there has been a focus on vaccinating children with special health care needs, with researchers studying their vaccination rates [12,13] and factors associated with their vaccine hesitancy [14-16]. Expert groups from countries such as the United States, Australia [17], and China [18] have developed guidelines and consensus to improve vaccination rates for children with special health care needs.

The global COVID-19 pandemic has heightened vaccine awareness [19]. China approved emergency use of inactivated COVID-19 vaccines for children aged 3 to 17 years in July 2021 [20]. Media focus on vaccination during the pandemic has shifted public and caregivers’ attitudes toward vaccines [21,22]. A survey across 6 countries showed that the pandemic has encouraged families who previously avoided vaccines to consider influenza vaccination [23]. A meta-analysis showed an increased willingness among caregivers to vaccinate their children against seasonal flu during the COVID-19 pandemic [24]. Caregivers of children with special health care needs may be more anxious than the general population [25]. The heightened public awareness of vaccines may lead to potential changes in vaccination attitudes among caregivers of children with special health care needs. However, there is a notable lack of research addressing vaccine hesitancy within this specific population.

In 2016, the Health Commission of Zhejiang province, China, set up the Vaccination Consultation Clinic at the Children’s Hospital, Zhejiang University School of Medicine [26]. Specialists there have been advising on vaccinations for children with special health care needs. Therefore, we conducted this retrospective study to investigate the attitudes toward vaccines among caregivers of children with special health care needs and evaluate the changes in caregivers’ hesitancy toward vaccines before, during, and after the COVID-19 pandemic. In addition, we also aimed to identify associated factors, including the stages of the COVID-19 pandemic, for caregivers’ attitudes toward NIP and non-NIP vaccines.

## Methods

### Study Design and Data Collection

Since 2016, the children’s hospital where this study was conducted has established the sole vaccination consultation clinic dedicated to providing assessments and tailored vaccination recommendations for children with special health care needs. The majority of these children were referred by physicians from vaccination sites throughout the province. This hospital is a grade A tertiary children’s hospital located in Zhejiang province, primarily serving pediatric patients within the province. In 2019, it was designated as a National Clinical Research Center for Child Health, thereby broadening its patient base. Based on this clinic, we conducted a retrospective analysis of caregivers’ vaccination willingness monitoring data from May 18, 2017, to May 31, 2023. Data collection used paper questionnaires until March 31, 2021, followed by an electronic monitoring system from April 1, 2021, onward. Trained research assistants transferred data collected from paper questionnaires into an electronic database managed by EpiData software (The EpiData Association). A double data entry process was implemented to ensure consistency and accuracy.

### Ethical Considerations

This study was conducted according to the Declaration of Helsinki [27] and was approved by the medical ethics committee of the Children’s Hospital Zhejiang University School of Medicine (2025-IRB-0005-P-01). This study involved secondary analysis of existing anonymized data that posed no privacy risks to individuals. The need for informed consent was waived by the ethics committee of Children’s Hospital, Zhejiang University School of Medicine. No financial compensation was provided as the study did not involve direct participant interaction.

### Study Population

Data were collected from children with special health care needs who visited the Vaccination Consultation Clinic at Children’s Hospital, Zhejiang University School of Medicine between May 18, 2017, and May 31, 2023. For the purposes of this study, children with special health care needs are defined as children with a medical condition that has persisted or is anticipated to persist for 12 months or longer, meeting at least one of the following criteria: (1) a persistent need for prescribed medications, (2) a persistent need for medical care that is higher than the average for children at the same age, (3) a persistent need for special treatments, (4) limitations on activities that are available for the majority of children at the same age, and (5) the presence of a persistent behavioral or developmental condition that requires treatment or counseling [26,28]. In total, 7770 children with special health care needs were included in this study.

### Measurements

Information on demographics, family history of diseases, history of adverse events following immunization, parental education levels, parental attitudes toward vaccination, and reasons for being referred to our Vaccination Consultation Clinic were extracted from the immunization evaluation system for children with special health care needs. Concurrently, medical records, such as the specific diagnosis of health conditions, were obtained from the hospital’s electronic medical records system.

We used 2 questions to measure caregivers’ hesitation toward NIP and non-NIP vaccines. The first question is “What is your attitude toward NIP vaccines?” There are three options: (1) I would like to choose NIP vaccines for my child; (2) if there are alternatives to the NIP vaccine (ie, alternative non-NIP vaccines), I am willing to pay and choose the alternative non-NIP vaccines for my child; and (3) I am not sure and hesitating to choose the NIP vaccines. If caregivers chose the option (3), we defined it as “hesitation toward NIP vaccines.” The second question is “What is your attitude toward the non-NIP vaccines?” There are two options: (1) I would like to choose non-NIP vaccines for my child and (2) I am not sure and hesitating. If caregivers chose the option (2), we defined it as “hesitation toward non-NIP vaccines.”

Based on the timeline of the COVID-19 pandemic in Zhejiang province, China, we defined stage I, II, and III. More specifically, stage I was “before the COVID-19 pandemic,” referred the time period from May 18, 2017, when the first questionnaire was filled to January 22, 2020; stage II was “during the COVID-19 pandemic,” referred from January 23, 2020, to December 18, 2022, when all the COVID-19 measures were eased in Zhejiang province; and stage III as from December 19, 2022, to May 31, 2023.

### Statistical Analysis

First, we conducted a descriptive analysis to present the general and clinical characteristics of the study population. Normality was tested for the continuous variable, that is, children’s age in our study. The distribution of age was not normal, so we calculated the median and IQR. Categorical variables were presented as numbers and percentages. In addition, we compared the difference in the general characteristics of the study population across 3 stages of the COVID-19 pandemic. Regarding the nonnormally distributed variable, we used the Kruskal-Wallis rank test, while for categorical variables, chi-square tests were applied. Furthermore, we described the spectrum of disease for all children enrolled in this study across the 3 phases of the COVID-19 pandemic. In addition, we described the disease spectrum of children with special health care needs in the overall population and the subgroup of each stage of the COVID-19 pandemic. Second, we described the percentages of caregivers’ choices for vaccines at 3 stages of the COVID-19 pandemic and compared the differences in the percentages of choices across 3 stages using chi-square tests. Finally, multinomial logistic regression models were used to identify independent variables that were associated with caregivers’ hesitation toward NIP and non-NIP vaccines. In addition, we conducted separate logistic regression analyses for different stages of the COVID-19 pandemic.

All statistical analyses were conducted using the R software (version 4.3.1; R Foundation for Statistical Computing). The statistical significance was indicated when *P*<.05 (2 tails).

## Results

### General and Clinical Characteristics of the Study Population

Table 1 presents the general characteristics of the study population. In total, 4418 (56.9%) children included in the analysis were male, and 3534 (45.5%) were aged less than 6 months. A total of 6051 (77.9%) questionnaires were filled in by mothers. Mothers of 3061 (39.4%) children had an educational level of college or above, while fathers of 3004 (38.7%) children had an educational level of college or above. There were statistically significant differences in children’s age, caregiver who filled in the questionnaire, and maternal and paternal educational level across the 3 COVID-19 pandemic stages (all *P*<.001).

**Table 1.** General characteristics of children and their caregivers (N=7770).

Items	Overall	Stage I^a^	Stage II^b^	Stage III^c^	*P* value
**Child’s sex, n (%)**					.50
Male	4418 (56.9)	1001 (56.8)	3108 (56.6)	309 (59.3)	
Female	3352 (43.1)	761 (43.2)	2379 (43.4)	212 (40.7)	
Age of the child (months), median (IQR)	7.0 (2.4-24.1)	6.4 (2.3-19.6)	7.6 (2.5-25.7)	5.3 (2.0-18.2)	<.001
**Age group of children (months), n (%)**					<.001
0‐6	3534 (45.5)	845 (48)	2416 (44)	273 (52.4)	
7‐12	1228 (15.8)	306 (17.4)	840 (15.3)	82 (16.7)	
13‐24	1053 (13.6)	235 (13.3)	755 (13.8)	63 (12.1)	
25‐72	1376 (17.7)	305 (17.3)	1001 (18.2)	70 (13.3)	
≥72	579 (7.4)	71 (4)	475 (8.7)	33 (6.3)	
**Caregiver who filled in the questionnaire, n (%)**					<.001
Mother	6051 (77.9)	1484 (84.2)	4208 (76.7)	359 (68.9)	
Father	1419 (18.3)	242 (13.7)	1049 (19.1)	128 (24.6)	
Others	281 (3.6)	33 (1.9)	214 (3.9)	34 (6.5)	
Missing	19 (0.2)	3 (0.2)	16 (0.3)	0 (0)	
**Maternal educational level, n (%)**					<.001
Middle school or below	1253 (16.1)	316 (17.9)	883 (16.1)	54 (10.4)	
High school or equivalent	1233 (15.9)	317 (18)	855 (15.6)	61 (11.7)	
Two-year college or associate degree	1948 (25.1)	463 (26.3)	1378 (25.1)	107 (20.5)	
Bachelor degree or above	3061 (39.4)	618 (35.1)	2209 (40.3)	234 (44.9)	
Missing	275 (3.5)	48 (3)	162 (3)	65 (12.5)	
**Paternal educational level, n (%)**					<.001
Middle school or below	1205 (15.5)	302 (17.1)	850 (15.5)	53 (10.2)	
High school or equivalent	1401 (18)	358 (20.3)	978 (17.8)	65 (12.5)	
Two-year college or associate degree	1796 (23.1)	435 (24.7)	1244 (22.7)	117 (22.5)	
Bachelor degree or above	3004 (38.7)	611 (34.7)	2170 (39.6)	223 (42.8)	
Missing	364 (4.7)	56 (3.2)	245 (4.5)	63 (12.1)	

a Stage Ⅰ was defined as the time period from May 18, 2017, to January 22, 2020.

b Stage II was defined as the time period from January 23, 2020, to December 18, 2022.

c Stage III was defined as the time period from December 19, 2022, to May 31, 2023.

Table 2 demonstrates the clinical characteristics of the study population. A total of 2729 (35.1%) children had comorbidity, 4739 (61%) children had a history of allergies, and 330 (4.2%) children had a history of adverse events following immunization. Regarding the reasons to visit the vaccination consultation clinic, 5245 (67.5%) caregivers reported that they were referred by doctors at the vaccination site, and 2198 (28.3%) caregivers came to consult because they did not know whether children with special health care needs can receive vaccines. In total, 336 (4.3%) caregivers came to consult because they did not know whether certain medications applied to the child may affect vaccination, and 130 (1.7%) caregivers due to adverse events in previous vaccinations. In addition, 288 (3.7%) caregivers were referred by the specialists because they were not sure about the vaccination of children with special health care needs, and 376 (4.8%) caregivers came to consult because their children were mandatory to be vaccinated in order to go to school.

**Table 2.** Clinical characteristics of the study population (N=7770).

Items	Overall, n (%)	Stage I^a^, n (%)	Stage II^b^, n (%)	Stage III^c^, n (%)
**Having comorbidity**				
Yes	2729 (35.1)	592 (66.4)	1915 (34.9)	222 (42.6)
No	5041 (64.9)	1170 (33.6)	3572 (65.1)	299 (57.4)
**History of allergy**				
Yes	4739 (61)	446 (25.3)	3772 (68.7)	521 (100)
No	3026 (38.9)	1316 (74.7)	1710 (31.2)	0 (0)
Missing	5 (0.1)	0 (0)	5 (0.1)	0 (0)
**History of AEFI** ^d^				
Yes	330 (4.2)	81 (5)	227 (4.1)	22 (4.2)
No	7358 (94.7)	1681 (95.4)	5178 (94.4)	499 (95.8)
Missing	82 (1.1)	0 (0)	82 (1.5)	0 (0)
**Reasons for visiting the vaccination consultation clinic**				
Referred by doctors at the vaccination site	5245 (67.5)	1407 (79.8)	3513 (64)	325 (62.4)
Caregivers came to consult because they did not know whether children with special health care needs can receive vaccines	2198 (28.3)	289 (16.4)	1716 (31.3)	193 (37)
Caregivers came to consult because they did not know whether certain medications applied to the child may affect vaccination	336 (4.3)	8 (0.4)	275 (5)	53 (10.2)
Caregivers came to consult because of adverse events in previous vaccinations	130 (1.7)	5 (0.3)	106 (1.9)	19 (3.6)
Referred by the specialist because they were not sure about vaccination of children with special health care needs	288 (3.7)	15 (0.8)	264 (4.8)	9 (1.7)
Required by school entry	376 (4.8)	13 (0.7)	300 (5.5)	63 (12.1)
Missing	86 (1.1)	25 (1.4)	58 (1.1)	3 (0.6)

a Stage Ⅰ was defined as the time period from May 18, 2017, to January 22, 2020.

b Stage II was defined as the time period from January 23, 2020, to December 18, 2022.

c Stage III was defined as the time period from December 19, 2022, to May 31, 2023.

d AEFI: adverse events following immunization.

### Disease Spectrum of the Study Population

Multimedia Appendix 1 shows the spectrum of disease in the overall study population and at each stage of the COVID-19 pandemic. The 3 most common diseases of those children with special health care needs were circulatory system diseases, nervous system diseases, and neonatal diseases in the overall population, and this pattern remains the same at each stage.

### Caregivers’ Attitude Toward NIP Vaccines and Non-NIP Vaccines

**Figure 1.**
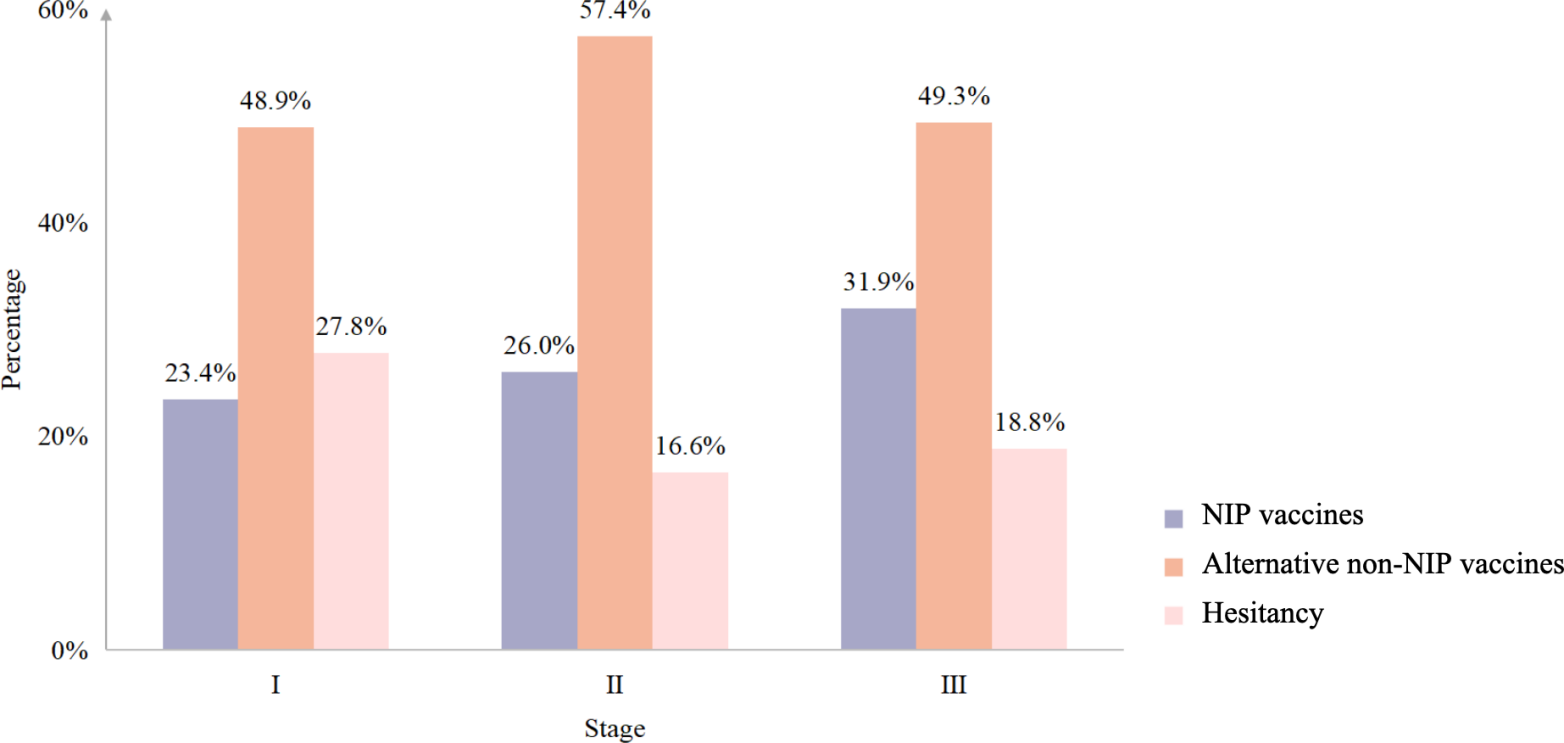
Caregivers’ choices for NIP and alternative non-NIP vaccines and their hesitancy at 3 stages of the COVID-19 pandemic. NIP: National Immunization Program.

**Figure 2.**
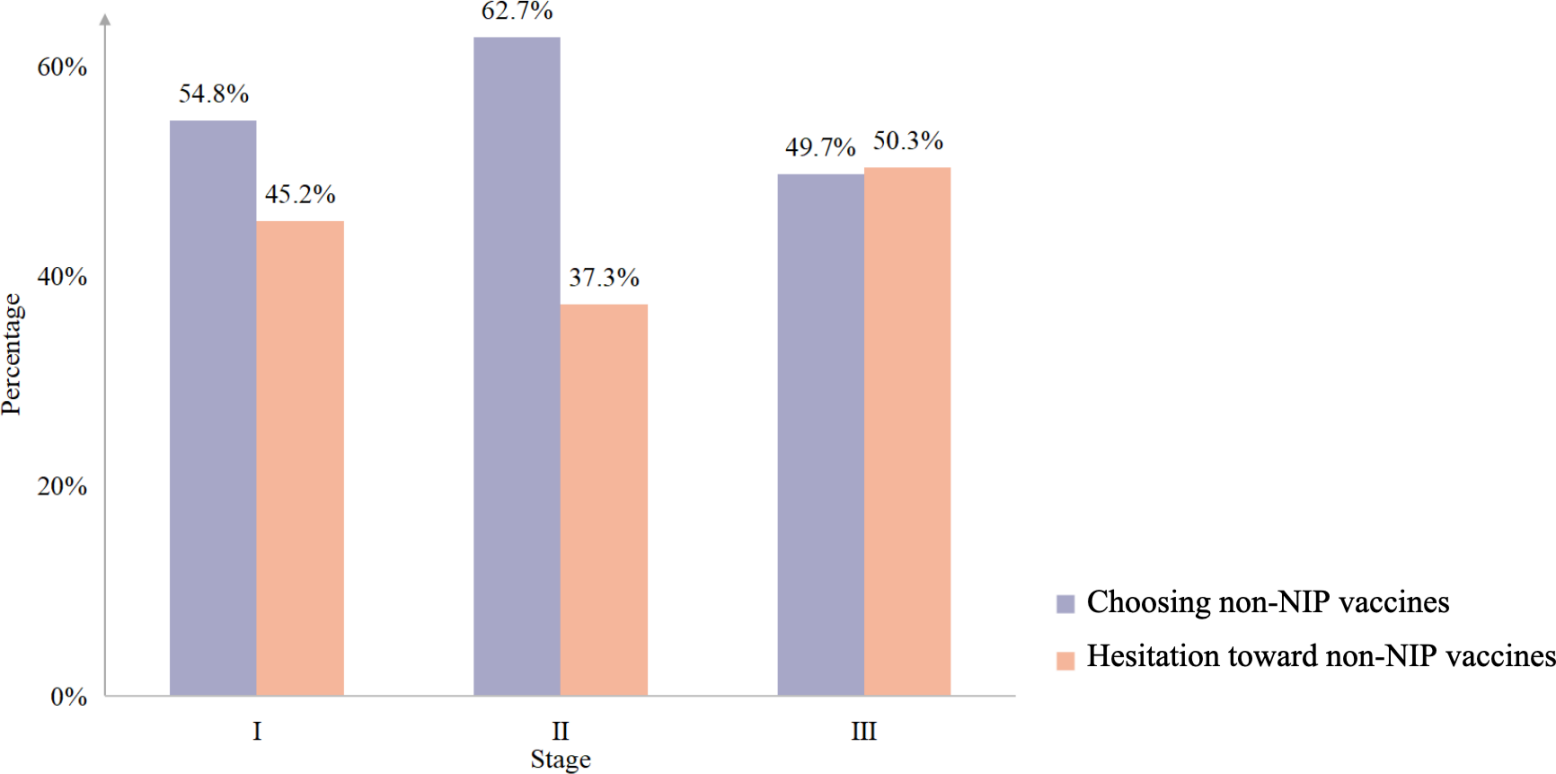
Caregivers’ choices for non-NIP vaccines and their hesitancy at 3 stages of the COVID-19 pandemic. NIP: National Immunization Program.

Figure 1 shows caregivers’ choice for NIP vaccines or alternative non-NIP vaccines and their hesitancy at each stage of the COVID-19 pandemic. Figure 2 shows caregivers’ choice of non-NIP vaccines and their hesitation at 3 stages of the COVID-19 pandemic. During the COVID-19 pandemic (ie, stage II), the percentages of choosing alternative non-NIP or non-NIP vaccines are highest (n=3148, 57.4% and n=3442, 67.7%, respectively) than those at the other 2 stages, while the percentages of caregivers’ hesitation toward NIP and non-NIP vaccines are lowest (n=911, 16.6% and n=2045, 37.3%, respectively). The percentages of caregivers’ attitudes toward NIP vaccines significantly differ across 3 stages of the COVID-19 pandemic (*P*<.001; Table S1 in Multimedia Appendix 2). The percentages of caregivers’ attitudes toward non-NIP vaccines significantly differ across 3 stages of the COVID-19 pandemic (*P*<.001; Table S2 in Multimedia Appendix 2).

### Associated Factors for Caregivers’ Choices of NIP Vaccines and Alternative Non-NIP Vaccines and Hesitations Toward NIP Vaccines

Table 3 presents the results of multinomial logistic regression regarding the associated factors for caregivers’ choices of alternative non-NIP vaccines and hesitations toward NIP vaccines using caregivers’ choices for NIP vaccines as a reference.

**Table 3.** Associated factors for caregivers’ hesitation toward NIP^a^ vaccines using multinomial logistic regression analysis.

Variables	Choosing alternative non-NIP vaccines, OR^b^ (95% CI)	Hesitation toward NIP vaccines, OR (95% CI)
**Stage of COVID-19 pandemic**		
I	Reference	Reference
II	1.3 (1.2-1.6)^c^	0.6 (0.5-0.8)^c^
III	0.9 (0.7-1.2)	0.6 (0.4-0.8)^d^
**Children’s sex**		
Male	Reference	Reference
Female	0.8 (0.8-1.0)^d^	0.9 (0.8-1.1)
**Age groups of children (months)**		
0‐6	Reference	Reference
7‐12	1.1 (0.9-1.3)	0.6 (0.5-0.7)^c^
13‐24	0.8 (0.7-1.0)	0.4 (0.3-0.5)^c^
25‐72	0.7 (0.6-0.8)^c^	0.4 (0.3-0.5)^c^
≥72	0.4 (0.3-0.5)^c^	0.5 (0.4-0.6)^c^
**Caregivers who filled the questionnaire**		
Mother	Reference	Reference
Father	0.8 (0.7-0.9)^c^	1.1 (0.9-1.3)
Others	1.3 (0.9-1.8)	2.4 (1.6-3.4)^c^
**Maternal educational level**		
Middle school or below	Reference	Reference
High school or equivalent	1.2 (1.0-1.5)	1.1 (0.8-1.4)
Two-year college	1.5 (1.2-1.8)^d^	1.2 (0.9-1.5)
Bachelor degree or above	2.3 (1.8-3.0)^c^	1.3 (1.0-1.8)
**Paternal educational level**		
Middle school or below	Reference	Reference
High school or equivalent	1.1 (0.9-1.3)	1.0 (0.8-1.3)
Two-year college	1.5 (1.2-1.9)^d^	1.0 (0.7-1.3)
Bachelor degree or above	1.4 (1.1-1.8)^d^	0.9 (0.7-1.2)
**Having comorbidity**		
No	Reference	Reference
Yes	1.0 (0.9-1.2)	1.2 (1.0-1.4)^e^
**History of allergy**		
No	Reference	Reference
Yes	0.6 (0.5-0.7)^c^	0.7 (0.6-0.8)^c^
**History of AEFI^f^**		
No	Reference	Reference
Yes	1.2 (0.9-1.5)	0.8 (0.5-1.1)

a NIP: National Immunization Program.

b OR: odds ratio.

c *P*<.001.

d *P*<.01.

e *P*<.05.

f AEFI: adverse events following immunization.

### Choosing Alternative Non-NIP Vaccines Versus NIP Vaccines

Stage II of the COVID-19 pandemic (*P*<.001), maternal educational level of 2-year college (*P*=.002) and of bachelor degree or above (*P*<.001), as well as paternal educational level of 2-year college (*P*=.001) and bachelor degree or above (*P*=.010) were significantly associated with higher odds to choose alternative non-NIP vaccines. In contrast, the child being female (*P*=.005), the child’s age of 25‐72 months (*P*<.001) and older than 72 months (*P*<.001), fathers filling in the questionnaire (*P*<.001), and having a history of allergy (*P*<.001) were significantly associated with lower odds of choosing alternative non-NIP vaccines.

### Hesitation Toward NIP Vaccines Versus Choosing NIP Vaccines

Caregivers other than parents filling in the questionnaire (*P*<.001) and having comorbidity (*P*=.02) were significantly associated with higher odds of hesitations toward NIP vaccines. Stage II (*P*<.001) and III (*P*=.003) of the COVID-19 pandemic, the child age older than 6 months (*P*<.001) and having a history of allergy (*P*<.001) were significantly associated with lower odds of hesitations toward NIP vaccines.

### Associated Factors for Caregivers’ Choices of Non-NIP Vaccines and Hesitations Toward Non-NIP Vaccines

Table 4 presents the results of multinomial logistic regression regarding the associated factors for caregivers’ hesitations toward non-NIP vaccines using the willingness of choosing non-NIP vaccines as a reference. The child age older than 72 months (*P*<.001), father (*P*<.001) and caregivers other than parents (*P*=.006) filling in the questionnaire, having comorbidity (*P*=.03), and having a history of allergy (*P*<.001) were significantly associated with higher odds of hesitation toward non-NIP vaccines. Stage II of the COVID-19 pandemic (*P*<.001), the child age of 7-12 months (*P*=.01), 13-24 months (*P*=.01), 25‐72 months (*P*=.001), maternal educational level of bachelor degree or above (*P*=.001) and paternal educational level of 2-year college (*P*=.002), and bachelor degree or above (*P*=.003) were significantly associated with lower odds of hesitation toward the non-NIP vaccines.

**Table 4.** Associated factors for caregivers’ hesitation toward non-NIP^a^ vaccines using multinomial logistic regression analysis.

Predictors	Hesitation toward non-NIP vaccines, OR^b^ (95% CI)
**Stage of COVID-19 pandemic**	
I	Reference
II	0.6 (0.5-0.7)^c^
III	1.0 (0.8-1.3)
**Children’s sex**	
Male	Reference
Female	1.1 (1.0-1.2)
**Age groups of children (months)**	
0‐6	Reference
7‐12	0.8 (0.7-1.0)^d^
13‐24	0.8 (0.7-1.0)^d^
25‐72	0.8 (0.7-0.9)^c^
≥72	1.6 (1.3-1.9)^c^
**Caregivers who filled the questionnaire**	
Mother	Reference
Father	1.6 (1.4-1.9)^c^
Others	1.5 (1.1-1.9)^e^
**Maternal educational level**	
Middle school or below	Reference
High school or equivalent	0.9 (0.8-1.1)
Two-year college	0.8 (0.7-1.0)
Bachelor degree and above	0.7 (0.6-0.8)^c^
**Paternal educational level**	
Middle school or below	Reference
High school or equivalent	1.0 (0.8-1.2)
Two-year college	0.7 (0.6-0.9)^e^
Bachelor degree and above	0.7 (0.6-0.9)^e^
**Having comorbidity**	
No	Reference
Yes	1.1 (1.0-1.2)^d^
**History of allergy**	
No	Reference
Yes	1.3 (1.2-1.4)^c^
**History of AEFI^f^**	
No	Reference
Yes	0.8 (0.7-1.1)

a NIP: National Immunization Program.

b OR: odds ratio.

c *P*<.001.

d *P*<.05.

e *P*<.01.

f AEFI: adverse events following immunization.

In addition, we presented the results of the separate logistic regression analysis for different stages of the COVID-19 pandemic in Tables S3-S8 in Multimedia Appendix 2.

## Discussion

### Principal Findings

This study used a large-scale, retrospective dataset to examine shifts in caregivers’ hesitancy of choosing NIP and non-NIP vaccines for children with special health care needs across the pre-, during, and postpandemic periods of COVID-19. Furthermore, we identified factors associated with vaccine hesitancy, including the different phases of the COVID-19 pandemic. To the best of our knowledge, this study has contributed to provide real-world evidence and comprehensive data on vaccine hesitancy among caregivers of children with special health care needs, addressing a gap in the existing literature.

Our findings indicate that, irrespective of the stage of the COVID-19 pandemic, the proportion of individuals opting for alternative non-NIP vaccines surpassed those choosing NIP vaccines. This trend may be attributed to the unique characteristics of the population under study, that is, children with special health care needs. These children differ from the healthy, general pediatric population, as they are managing certain health conditions. Consequently, their caregivers may have greater caution when selecting vaccines. The alternative non-NIP vaccines available are either inactivated vaccines, as opposed to live-attenuated NIP vaccines, or vaccines with a higher number of valences. It is understandable that caregivers opt for inactivated vaccines over live-attenuated vaccines to mitigate the potential side effects associated with live pathogens. Furthermore, parents may prefer to administer vaccines with multiple valences in a single injection to their child.

Our study revealed that caregivers’ acceptance of NIP vaccines increased across the pre-, during, and postpandemic periods of COVID-19. Wang et al [29] also found that there has been an increasing acceptance of routine childhood vaccination and COVID-19 vaccination from 2020 to 2021 via a repeated cross-sectional survey. We found that the proportion of caregivers of children with special health care needs willing to vaccinate their children with alternative non-NIP vaccines was highest during the pandemic. Concurrently, we observe the lowest proportion of caregivers exhibiting hesitation toward NIP vaccines at the same stage. These findings align with the results of a multinomial logistic analysis, which indicated that this stage (ie, during the pandemic) is associated with significantly higher odds of caregivers’ willingness to select alternative non-NIP vaccines. Additionally, stages II and III are significantly associated with lower odds of caregivers’ hesitation toward NIP vaccines. Zhang et al [30] reported that the coverage rate of non-NIP vaccines increased by 25.8% and 34.7%, respectively, in 2020 and 2021 when it was the nonpharmaceutical intervention period compared to the prepandemic period (ie, in 2019). Our finding also echoed with previous conclusion that the COVID-19 pandemic has caused unprecedented impacts including parental trust in vaccines [31]. During the COVID-19 pandemic, both the government and the mass media have substantially intensified their advocacy and promotion of the COVID-19 vaccine. They have also enhanced risk communication to raise awareness about the benefits and risks of vaccination and to promote the scientific belief that the benefits outweigh the risks during the COVID-19 pandemic [32]. This heightened publicity has concurrently enhanced public awareness regarding vaccines in general, potentially fostering a more positive attitude and greater acceptance of vaccines among the population. Consequently, caregivers have become more inclined to vaccinate their children during the pandemic; they are even willing to pay for the self-funded, alternative NIP vaccines.

Regarding caregivers’ attitudes toward non-NIP vaccines, our study indicates that caregivers’ willingness to vaccinate their children with special health care needs was at its peak, and their hesitancy was at its lowest during the pandemic. This observation aligns with the results obtained from the multinomial regression analysis. The increased willingness can likely be attributed to the extensive promotion of vaccines, particularly the COVID-19 vaccine, by governments and social media platforms. However, following the COVID-19 pandemic, our study observed a decline in the proportion of caregivers opting for non-NIP vaccines and an increase in their hesitancy toward these vaccines. In this study, stage III corresponds to the 6-month period following the relaxation of COVID-19 measures in China, during which the Chinese public exhibited a varied and mixed attitude toward vaccines. This period was characterized by both trust and mistrust in vaccines, exacerbated by reports of incidents such as “fake vaccines” or vaccine scandals that were disseminated through social media. There has been a scarcity of literature regarding the impact of the COVID-19 pandemic on non-NIP vaccination intention for children. Wang et al [33] found that in a community sample, approximately 70% of parents did not change their intention for self-paid, non-NIP vaccines for their children, and about 20% of parents increased their intention, while about 10% decreased the intention after the COVID-19 pandemic. In this study, a short period of stage III resulted in a limited sample size for data collection, potentially leading to findings that may be attributable to chance. Therefore, we recommend conducting future studies with an increased sample size by extending the study period during the “postpandemic” phase to validate or refute our findings.

Our study indicated a sex disparity in caregivers’ selection of alternative, self-funded non-NIP vaccines. Caregivers of female children were less likely to choose these alternative non-NIP vaccines compared to caregivers of male children. This pattern remained the same before and during the pandemic but was reversed after the pandemic when caregivers of female children were more likely to choose the alternative non-NIP compared to caregivers of male children. Although global immunization coverage does not show significant differences between male and female children, certain countries and communities may experience sex-related barriers to immunization due to prevailing social and cultural norms [34]. The outbreak of the COVID-19 pandemic might have increased the intention of caregivers of female children to choose paid alternative non-NIP vaccines for them.

Regarding the age of children, our study indicated that caregivers of older children demonstrated a lower propensity to vaccinate their children with alternative non-NIP vaccines or exhibited reduced vaccine hesitancy compared to caregivers of very young children (ie, 0‐6 months). This pattern remains the same in the separate logistic analysis in each stage. Notably, our findings revealed that the likelihood of vaccine hesitancy toward non-NIP vaccines among caregivers of children older than 72 months was 1.58 times greater than that among caregivers of children aged 0‐6 months. A potential experiential factor may account for this finding. Non-NIP vaccines are not mandatory for children, and advocacy or public awareness efforts are less robust compared to those for NIP vaccines. Advocacy predominantly targets young children, typically younger than 6 years of age. Consequently, caregivers of older children may lack awareness regarding the appropriate timing and types of non-NIP vaccines beneficial for their children. Therefore, our study suggests that it is important and necessary to educate caregivers of children older than 6 years.

In this study, our findings indicate that fathers, in comparison to mothers, exhibited a lower propensity to choose for alternative non-NIP vaccines and demonstrated a greater likelihood of hesitancy in selecting non-NIP vaccines for their children. Furthermore, the analysis revealed that caregivers other than parents showed a higher probability of hesitancy in choosing non-NIP vaccines for children when compared to mothers. This finding is noteworthy and may be attributed to the cultural norm within Chinese families, where mothers predominantly assume the role of primary caregiver and are chiefly responsible for making medical decisions concerning their children. While it is important to acknowledge the contributions of fathers and other caregivers in child-rearing, their involvement and roles may not be as prominent or comparable to those of mothers.

Our study demonstrates that a relatively high maternal and paternal educational level, such as a 2-year college and bachelor or above degree, is associated with the higher intention for choosing alternative non-NIP vaccines and less hesitation to both NIP and non-NIP vaccines, which is consistent with previous studies. For instance, a study in Shanghai showed that higher income, higher education, and greater access to vaccines made respondents more willing to immunize their children with non-NIP [35]. Economic status remained a significant factor in determining the acceptability of non-NIP vaccines [36]. Parents’ high education is usually linked to high health literacy, which greatly affects their ability to use health information to make health decisions for their children [37].

In this study, caregivers of children with comorbidities exhibit increased hesitancy regarding NIP and non-NIP vaccines. This hesitancy is understandable, as the existing health conditions of these children already present significant concerns, prompting caregivers to exercise greater caution when considering vaccination decisions. We also found that caregivers of children with allergy had increased hesitancy regarding non-NIP vaccines, which may stem from concerns about an elevated risk of adverse effects following vaccination in children with a history of allergies.

The vaccination of children with special health care needs is unique, as their health issues may lead to delayed or missed vaccinations, leaving them vulnerable to infections. We analyzed how China’s macroprevention and control strategies for COVID-19 may affect the vaccination intentions of this group using a large sample. Our findings indicate that during the COVID-19 pandemic, caregivers exhibited a marked increase in their willingness to receive vaccinations, accompanied by a significant decrease in vaccine hesitancy, compared to levels observed both prior to and following the pandemic. This empirical conclusion aligns with observations made by physicians in vaccination clinics. The onset of the pandemic appears to have heightened parental awareness regarding vaccines, thereby enhancing vaccination rates. This study revealed that a significant proportion of parents continue to exhibit hesitancy toward vaccines, highlighting the persistence of a knowledge gap and the potential for enhancement in health education initiatives. Furthermore, the research identified several factors associated with vaccine hesitancy, including the child’s age and sex, the educational level of the parents, and the presence of an allergy history. These findings aim to furnish evidence-based support for the formulation of personalized and targeted health education strategies in future endeavors. The findings of this study further indicate the necessity for social mobilization from a macrolevel perspective to collaboratively enhance vaccine awareness and advance health equity.

### Strengths and Limitations

To the best of our knowledge, this study is the first to use a large sample size (N=7770) to examine attitudes, specifically hesitancy, toward vaccinations among caregivers of children with special health care needs before, during, and after the COVID-19 pandemic. Additionally, our study has documented a comprehensive set of variables, which allows us to apply multivariate regression analysis to identify associated factors for caregivers’ attitudes toward NIP and non-NIP vaccines in China. We consider it to be among the few studies of its kind conducted globally.

However, several limitations merit consideration. First, this study used a cross-sectional design, which restricts our ability to infer causality and confines our analysis to assessing associations. Second, we did not use a standardized or validated instrument, such as the Vaccine Hesitancy Scale, to measure vaccine hesitancy. Instead, we used 2 self-developed questions as a proxy for assessing vaccine hesitancy. Third, our study exclusively assessed caregivers’ willingness or attitudes toward vaccinating their children with NIP or non-NIP vaccines. It is important to acknowledge the potential discrepancy between the respondents’ self-reported willingness to vaccinate and their actual vaccination behavior, as survey responses may not always accurately reflect real-world actions [38].

### Conclusions

This study demonstrated that caregivers’ willingness to vaccinate their children with special health care needs with NIP and non-NIP vaccines was highest and their hesitancy was lowest during the COVID-19 pandemic in China. Additionally, we have identified multiple factors associated with caregivers’ willingness and hesitancy of vaccinating their children. These findings provide evidence-based support for developing personalized health education strategies and further indicate the necessity for social mobilization at a macrolevel to collaboratively enhance vaccine awareness and health literacy regarding vaccinating children with special health care needs.

## Supplementary material

10.2196/67487 Multimedia Appendix 1 The spectrum of disease in the overall study population and at each stage of the COVID-19 pandemic. (A) The spectrum of disease in the overall study population, (B) the spectrum of disease at stage I of the COVID-19 pandemic, (C) the spectrum of disease at stage II of the COVID-19 pandemic, and (D) the spectrum of disease at stage III of the COVID-19 pandemic.

10.2196/67487 Multimedia Appendix 2 Additional tables.

## Abbreviations

NIP: National Immunization Program
