# Supplementary figures and images for: Vaccine Hesitancy and Associated Factors Among Caregivers of Children With Special Health Care Needs in the COVID-19 Era in China: Cross-Sectional Study
Source: JMIR Public Health Surveill. 2025 Mar 26;11:e67487. doi: 10.2196/67487 (PMC11964954; doi:10.2196/67487)

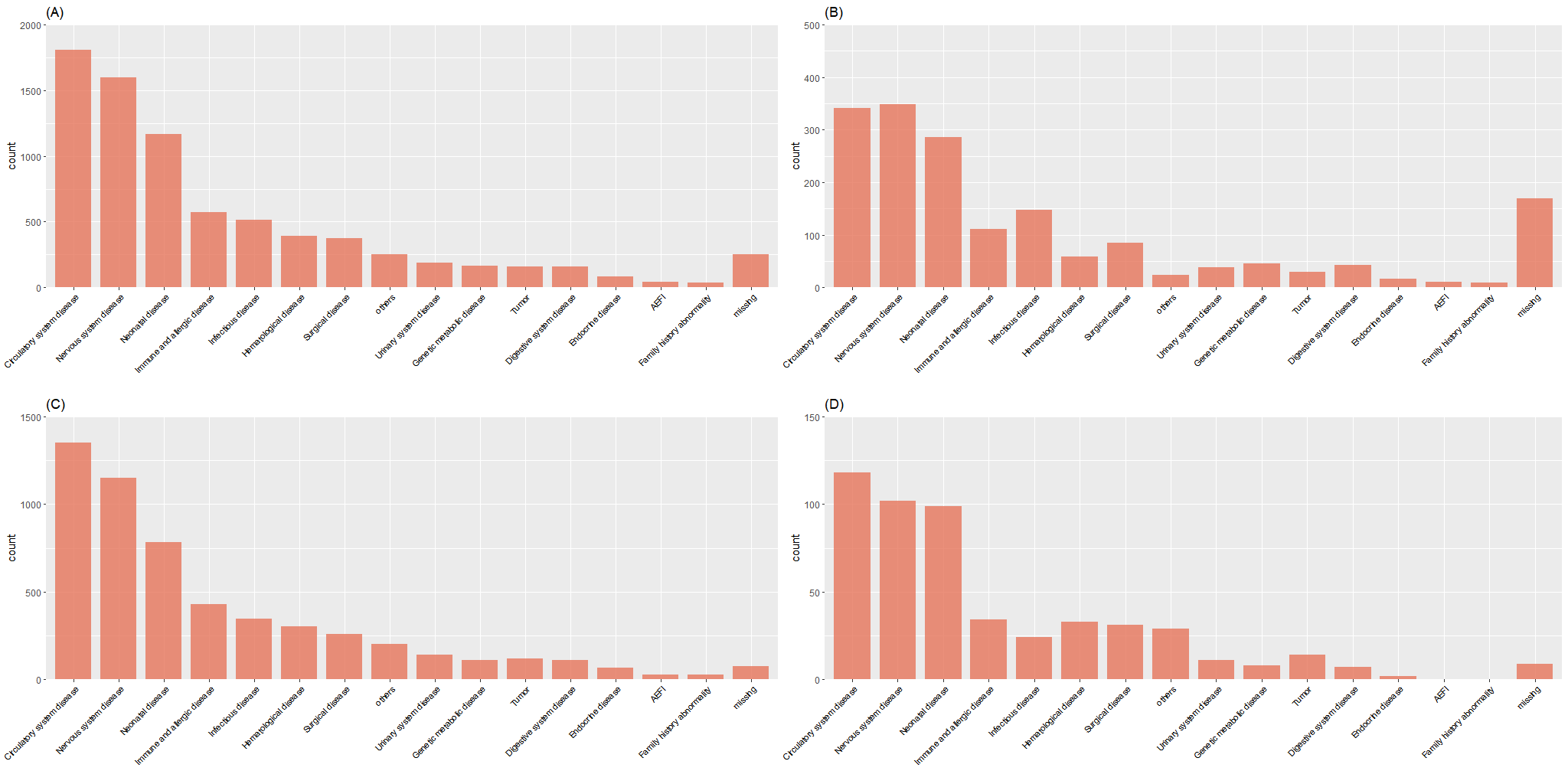

Supplement: Multimedia Appendix 1 [file publichealth-v11-e67487-s001.tiff]
