# Supplementary material for: Vaccine Hesitancy and Associated Factors Among Caregivers of Children With Special Health Care Needs in the COVID-19 Era in China: Cross-Sectional Study
Source: JMIR Public Health Surveill. 2025 Mar 26;11:e67487. doi: 10.2196/67487 (PMC11964954; doi:10.2196/67487)
Supplement: Multimedia Appendix 2 [file publichealth-v11-e67487-s002.docx]

Table S1. Comparison of caregivers’ attitudes towards NIP vaccines across three different stages of COVID-19 pandemic

| Choice | Stage I | Stage II | Stage III | P value |
| --- | --- | --- | --- | --- |
|  |  |  |  | <0.001 |
| Be willing to choose NIP vaccines | 412 (23.4) | 1428 (26.0) | 166 (31.9) |  |
| Be willing to choose alternative non-NIP vaccines | 861 (48.9) | 3148 (57.4) | 257 (49.3) |  |
| Hesitation towards NIP vaccines | 489 (27.8) | 911 (16.6) | 98 (18.8) |  |

Table S2. Comparison of caregivers’ attitudes towards non-NIP vaccines across three different stages of COVID-19 pandemic

|  | Stage I | Stage II | Stage III | P value |
| --- | --- | --- | --- | --- |
|  |  |  |  | <0.001 |
| Be willing to choose non-NIP vaccines | 965 (54.8) | 3442 (67.7) | 259 (49.7) |  |
| Hesitation towards non- NIP vaccines | 797 (45.2) | 2045 (37.3) | 262 (50.3) |  |

Table S3. Associated factors for caregivers’ hesitation towards NIP vaccines using multinomial logistic regression analysis before the COVID-19 pandemic

| Variables | Choosing alternative non-NIP vaccines | Hesitation towards NIP vaccines |
| --- | --- | --- |
| Children’s sex |  |  |
| Boy | reference | reference |
| Girl | 0.7 (0.5, 0.9) ^b^ | 0.8 (0.6, 1.0)^a^ |
| Age groups of children |  |  |
| 0-6 months | reference | Reference |
| 7-12 months | 1.1 (0.8, 1.6) | 0.5 (0.3, 0.7) ^c^ |
| 13-24 months | 0.6 (0.4, 0.9)^a^ | 0.3 (0.2, 0.5) ^c^ |
| 25-72 months | 0.9 (0.6, 1.1) | 0.3 (0.2, 0.4) ^c^ |
| ≥ 72 months | 0.4 (0.2, 0.7) ^b^ | 0.3 (0.2, 0.6) ^b^ |
| Caregivers who filled the questionnaire | | |
| Mother | reference | reference |
| Father | 1.2 (0.8, 1.7) | 1.1 (0.8, 1.7) |
| Others | 1.6 (0.6, 4.3) | 1.4 (0.4,4.7) |
| Maternal educational level |  |  |
| Middle school or below | reference | reference |
| High school or equivalent | 1.0 (0.6, 1.6) | 1.3 (0.8, 2.2) |
| Two-year College | 1.4 (0.8, 2.3) | 1.4 (0.8, 2.4) |
| Bachelor's degree or above | 2.3 (1.4, 4.0) ^b^ | 2.0 (1.1, 3.7)^a^ |
| Paternal educational level |  |  |
| Middle school or below | reference | reference |
| High school or equivalent | 1.6 (1.0, 2.5) | 1.3 (0.8, 2.1) |
| Two-year College | 1.9 (1.1, 3.1) ^a^ | 1.1 (0.6, 2.0) |
| Bachelor's degree or above | 1.7 (1.0, 3.0) | 1.0 (0.5, 1.8) |
| Having comorbidity |  |  |
| No | reference | reference |
| Yes | 1.2 (0.9, 1.6) | 1.4 (1.0, 1.8) ^a^ |
| History of allergy |  |  |
| No | reference | reference |
| Yes | 0.9 (0.7, 1.2) | 0.9 (0.6, 1.2) |
| History of AEFI |  |  |
| No | reference | reference |
| Yes | 0.9 (0.5, 1.5) | 0.5 (0.2, 1.1) |

^a^*P*<.05, ^b^*P*<.01, ^c^*P*<.001.

Table S4. Associated factors for caregivers’ hesitation towards NIP vaccines using multinomial logistic regression analysis during the COVID-19 pandemic

| Variables | Choosing alternative non-NIP vaccines | Hesitation towards NIP vaccines |
| --- | --- | --- |
| Children’s sex |  |  |
| Boy | reference | reference |
| Girl | 0.8 (0.7, 1.0) ^a^ | 0.9 (0.8, 1.1) |
| Age groups of children |  |  |
| 0-6 months | reference | Reference |
| 7-12 months | 1.1 (0.9, 1.3) | 0.6 (0.5, 0.8) ^c^ |
| 13-24 months | 0.9 (0.7, 1.1) | 0.4 (0.3, 0.6) ^c^ |
| 25-72 months | 0.6 (0.5, 0.7)^c^ | 0.4 (0.3, 0.5) ^c^ |
| ≥ 72 months | 0.4 (0.3, 0.5) ^c^ | 0.5 (0.4, 0.7) ^c^ |
| Caregivers who filled the questionnaire | | |
| Mother | reference | reference |
| Father | 0.7 (0.6, 0.8) ^c^ | 1.2 (1.0, 1.5) |
| Others | 1.3 (0.9, 1.8) | 2.4 (1.6, 3.7)^c^ |
| Maternal educational level |  |  |
| Middle school or below | reference | reference |
| High school or equivalent | 1.2 (1.0, 1.6) | 0.9 (0.7, 1.3) |
| Two-year College | 1.4 (1.1, 1.9)^a^ | 1.0 (0.7, 1.4) |
| Bachelor's degree or above | 2.2 (1.6, 3.0) ^c^ | 1.0 (0.7, 1.5) |
| Paternal educational level |  |  |
| Middle school or below | reference | reference |
| High school or equivalent | 1.0 (0.7, 1.2) | 1.1 (0.8, 1.5) |
| Two-year College | 1.4 (1.0, 1.8) ^a^ | 1.0 (0.7, 1.4) |
| Bachelor's degree or above | 1.4 (1.0, 1.8)^a^ | 1.0 (0.7, 1.5) |
| Having comorbidity |  |  |
| No | reference | reference |
| Yes | 1.0 (0.8, 1.2) | 1.17 (0.97, 1.42) |
| History of allergy |  |  |
| No | reference | reference |
| Yes | 0.6 (0.5, 0.7) ^c^ | 0.6 (0.5, 0.7) ^c^ |
| History of AEFI |  |  |
| No | reference | reference |
| Yes | 1.3 (0.9, 1.8) | 0.9 (0.6, 1.5) |

^a^*P*<.05, ^b^*P*<.01, ^c^*P*<.001.

Table S5. Associated factors for caregivers’ hesitation towards NIP vaccines using multinomial logistic regression analysis after the COVID-19 pandemic

| Variables | Choosing alternative non-NIP vaccines | Hesitation towards NIP vaccines |
| --- | --- | --- |
| Children’s sex |  |  |
| Boy | reference | reference |
| Girl | 1.7 (1.1, 2.7) ^a^ | 1.3 (0.7, 2.3) |
| Age groups of children |  |  |
| 0-6 months | reference | Reference |
| 7-12 months | 1.6 (0.9, 3.2) | 0.7 (0.3, 1.8) |
| 13-24 months | 1.3 (0.6, 2.8) | 0.9 (0.3, 2.3) |
| 25-72 months | 1.0 (0.5, 2.0) | 0.9 (0.4, 2.2 |
| ≥ 72 months | 0.7 (0.2, 1.8) | 0.7 (0.2, 2.3) |
| Caregivers who filled the questionnaire | | |
| Mother | reference | reference |
| Father | 1.0 (0.6, 1.7) | 1.3 (0.7, 2.5) |
| Others | 0.9 (0.2, 3.9) | 5.6 (1.4, 23.0)^b^ |
| Maternal educational level |  |  |
| Middle school or below | reference | reference |
| High school or equivalent | 1.6 (0.6, 4.4) | 2.0 (0.6, 6.2) |
| Two-year College | 1.6 (0.6, 4.4) | 1.6 (0.5, 5.1) |
| Bachelor's degree or above | 3.3 (1.1, 9.6) ^*^ | 3.3 (0.9, 11.5) |
| Paternal educational level |  |  |
| Middle school or below | reference | reference |
| High school or equivalent | 1.6 (0.6, 5.0) | 0.3 (0.1, 0.9)^a^ |
| Two-year College | 2.8 (0.9, 8.2) | 0.4 (0.1, 1.1) |
| Bachelor's degree or above | 1.7 (0.6, 5.4) | 0.2 (0.1, 0.6)^b^ |
| Having comorbidity |  |  |
| No | reference | reference |
| Yes | 0.8 (0.5, 1.3) | 1.0 (0.6, 1.8) |
| History of allergy |  |  |
| No | reference | reference |
| Yes | 0.6 (0.4, 0.9) ^*^ | 0.9 (0.6, 1.4) |
| History of AEFI |  |  |
| No | reference | reference |
| Yes | 0.7 (0.2, 2.0) | 0.4 (0.1, 2.3) |

^a^*P*<.05, ^b^*P*<.01, ^c^*P*<.001.

Table S6. Associated factors for caregivers’ hesitation towards non-NIP vaccines using binary logistic regression analysis before the COVID-19 pandemic

| Variables | Hesitation towards non-NIP vaccines |
| --- | --- |
| Children’s sex |  |
| Boy | Reference |
| Girl | 1.0 (0.8,1.3 ) |
| Age groups of children |  |
| 0-6 months | Reference |
| 7-12 months | 0.6 (0.4,0.8 ) ^c^ |
| 13-24 months | 0.8 (0.6,1.0 ) |
| 25-72 months | 0.5 (0.4,0.6 ) ^c^ |
| ≥ 72 months | 0.9 (0.5,1.5 ) |
| Caregivers who filled the questionnaire |  |
| Mother | Reference |
| Father | 1.3 (1.0,1.7 ) |
| Others | 1.4 (0.6,3.1 ) |
| Maternal educational level |  |
| Middle school or below | Reference |
| High school or equivalent | 1.3 (0.9,1.9 ) |
| Two-year College | 1.1 (0.8,1.7 ) |
| Bachelor's degree and above | 1.0 (0.6,1.5 ) |
| Paternal educational level |  |
| Middle school or below | Reference |
| High school or equivalent | 0.9 (0.6,1.3 ) |
| Two-year College | 0.6 (0.4,1.0 ) ^a^ |
| Bachelor's degree and above | 0.6 (0.4,0.9 ) ^a^ |
| Having comorbidity |  |
| No | Reference |
| Yes | 1.2 (0.9,1.4 ) |
| History of allergy |  |
| No | Reference |
| Yes | 1.0 (0.8,1.2 ) |
| History of AEFI |  |
| No | Reference |
| Yes | 0.6 (0.3,1.0 ) ^a^ |

^a^*P*<.05, ^b^*P*<.01, ^c^*P*<.001.

Table S7. Associated factors for caregivers’ hesitation towards non-NIP vaccines using binary logistic regression analysis during the COVID-19 pandemic

| Variables | Hesitation towards non-NIP vaccines |
| --- | --- |
| Children’s sex |  |
| Boy | Reference |
| Girl | 1.1 (1.0, 1.2) |
| Age groups of children |  |
| 0-6 months | Reference |
| 7-12 months | 1.0 (0.8, 1.1) |
| 13-24 months | 0.8 (0.6, 0.9) ^a^ |
| 25-72 months | 0.9 (0.7, 1.0) |
| ≥ 72 months | 1.8 (1.4, 2.2) ^c^ |
| Caregivers who filled the questionnaire |  |
| Mother | Reference |
| Father | 1.8 (1.5, 2.0) ^c^ |
| Others | 1.4 (1.0, 1.9) ^a^ |
| Maternal educational level |  |
| Middle school or below | Reference |
| High school or equivalent | 0.8 (0.6, 1.0) |
| Two-year College | 0.7 (0.6, 0.9)^a^ |
| Bachelor's degree and above | 0.6 (0.4, 0.8) ^c^ |
| Paternal educational level |  |
| Middle school or below | Reference |
| High school or equivalent | 1.0 (0.8, 1.3) |
| Two-year College | 0.8 (0.6, 1.0) |
| Bachelor's degree and above | 0.8 (0.6, 1.0) |
| Having comorbidity |  |
| No | Reference |
| Yes | 1.1 (0.9, 1.2) |
| History of allergy |  |
| No | Reference |
| Yes | 1.4 (1.2, 1.6) ^c^ |
| History of AEFI |  |
| No | Reference |
| Yes | 0.9 (0.7, 1.3) |

^a^*P*<.05, ^b^*P*<.01, ^c^*P*<.001.

Table S8. Associated factors for caregivers’ hesitation towards non-NIP vaccines using binary logistic regression analysis after the COVID-19 pandemic

| Variables | Hesitation towards non-NIP vaccines |
| --- | --- |
| Children’s sex |  |
| Boy | Reference |
| Girl | 0.9 (0.6, 1.4) |
| Age groups of children |  |
| 0-6 months | Reference |
| 7-12 months | 0.7 (0.4, 1.2) |
| 13-24 months | 1.8 (0.9, 3.5) |
| 25-72 months | 1.7 (0.9, 3.2) |
| ≥ 72 months | 1.5 (0.6, 3.5) |
| Caregivers who filled the questionnaire |  |
| Mother | Reference |
| Father | 1.4 (0.9, 2.2) |
| Others | 2.6 (0.8, 8.0) |
| Maternal educational level |  |
| Middle school or below | Reference |
| High school or equivalent | 1.0 (0.4, 2.6) |
| Two-year College | 1.3 (0.5, 3.3) |
| Bachelor's degree and above | 0.8 (0.3, 2.0) |
| Paternal educational level |  |
| Middle school or below | Reference |
| High school or equivalent | 0.6 (0.2, 1.5) |
| Two-year College | 0.5 (0.2, 1.2) |
| Bachelor's degree and above | 0.5 (0.2, 1.4) |
| Having comorbidity |  |
| No | Reference |
| Yes | 1.7 (1.1, 2.6) ^a^ |
| History of allergy |  |
| No | Reference |
| Yes | 1.1 (0.8, 1.6) |
| History of AEFI |  |
| No | Reference |
| Yes | 1.2 (0.4, 3.3) |

^a^*P*<.05.
